# Contextual and Individual Factors Influencing Periodontal Treatment Needs by Elderly Brazilians: A Multilevel Analysis

**DOI:** 10.1371/journal.pone.0156231

**Published:** 2016-06-01

**Authors:** Chaiane Emilia Dalazen, Alessandro Diogo De Carli, Rafael Aiello Bomfim, Mara Lisiane Moraes dos Santos

**Affiliations:** 1 Faculdade de Odontologia “Prof Albino Coimbra Filho”, Saúde Coletiva, Universidade Federal de Mato Grosso do Sul, Campo Grande, MS, Brazil; 2 Departamento de Tecnologia de Alimentos e Saúde Coletiva, Universidade Federal de Mato Grosso do Sul, Campo Grande, MS, Brazil; Boston University Goldman School of Dental Medicine, UNITED STATES

## Abstract

**Objective:**

To assess the relationship between periodontal treatment needs by elderly Brazilians and contextual as well as individual variables.

**Methods:**

A cross-sectional study was carried out to assess the need for clinical periodontal treatment, based on National Oral Health Survey (SB Brasil 2010) data on the presence of dental calculus, shallow (3–5 mm) and deep (≥ 6 mm) periodontal pockets, and gingival bleeding in elderly people (n = 7,619). The contextual variables included the Municipal Human Development Index (MHDI), income inequality (Gini Index) and coverage of the municipal population by the Family Health Strategy (FHS) program oral health teams.<0} The individual variables were sex, income, education level and self-reported skin color. Multilevel logistic regression models were used to calculate the odds ratio (OR) and 95% confidence intervals (CI95%) between periodontal treatment needs and the contextual as well as individual variables.

**Results:**

Gingival bleeding was found in 20.7% of the elderly analyzed (n = 1,577), dental calculus in 34% (n = 2,590), shallow periodontal pockets in 15.6% (n = 1,189), and deep periodontal pockets in 4.2% (n = 320). Individual factors were correlated with all the outcomes assessed. Sex was a protective factor in regard to gingival bleeding (OR = 0.87; CI95% 0.76–1.00), dental calculus (OR = 0.86; CI95% 0.75–0.99), shallow periodontal pockets (OR = 0.69; CI95% 0.60–0.80) and deep periodontal pockets (OR = 0.58; CI95% 0.45–0.74). It was found that fewer women needed treatment. Elderly people who self-reported having nonwhite skin had higher chances of needing periodontal treatment. Skin color was a risk factor for gingival bleeding (OR = 1.32; CI95% 1.14–1.53), dental calculus (OR = 1.32; CI95%1.14–1.54) and shallow periodontal pockets (OR = 1.27; CI95% 1.09–1.49). Education level was associated with the presence of dental calculus (OR = 0.77; CI95% 0.66–0.89), shallow periodontal pockets (OR = 0.86; CI95% 0.73–1.00) and deep periodontal pockets (OR = 0.74; CI95% 0.57–0.97), thus acting as a risk factor for undereducated elderly people. There was a correlation between population coverage by the Family Health Strategy (FHS) program oral health teams and the presence of gingival bleeding (OR = 0.67; CI95% 0.52–0.88), shallow periodontal pockets (OR = 0.76; CI95% 0.58–0.98) and deep periodontal pockets (OR = 0.62; CI95% 0.44–0.89), making these teams act as a protective factor.

**Conclusions:**

This study showed evidence of the sociocontextual as well as individual sociodemographic characteristics influencing periodontal treatment needed by elderly Brazilians, based on the clinical features of periodontal disease. The results suggest the existence of inequality related to periodontal treatment needs among elderly Brazilians, especially in regard to sex and ethnicity, in addition to a potentially positive impact from the expansion of the Family Health Strategy (FHS) program oral health teams.

## Introduction

The proportion of the elderly Brazilian population has increased owing to the demographic transition process that Brazil has been undergoing [1–3]. Changes in the age structure of the overall population affect the demands for health services, including dental services. This requires information to be gathered to aid in the planning of health services capable of satisfying these needs [4].

Oral disease is considered a major public health issue in several countries, considering that poor oral health conditions could produce not only systemic effects, but also a negative impact on an individual’s quality of life [5]. Periodontal disease is one of the most prevalent oral diseases. Its presence and severity have been associated with socially deprived populations, thus suggesting a correlation between periodontal disease and social inequality [6,7].

According to Sheiham and Netuveli [8], only a small percentage of adults in Europe are affected by severe periodontal disease, with more advanced stages of periodontal disease commonly identified among the elderly. Corbet, Zi and Lo [9] assessed studies on periodontal disease, published in Asia and Oceania, and concluded that both socioeconomic conditions and health systems have an influence on the periodontal conditions of the overall population. Similarly, Borrell et al. [10] conducted a study with the North American population, and found that socioeconomic conditions influence the prevalence of periodontitis among elderly people. In Japan, periodontal disease was considered the main cause behind extractions among patients older than 45 years of age [11].

In Brazil, extractions resulting from avoidable diseases, such as periodontal disease, lead to a high rate of edentulism among elderly Brazilians [12–14] Therefore, it is of paramount importance to identify the need for treatment among the elderly and the factors influencing this need, particularly because this knowledge could be applied to reduce inequality and thus allow unrestricted access to treatment [15] This would make it possible to preserve dental structures, and prevent the worsening of systemic diseases, such as cardiovascular disorders and diabetes mellitus [16]. These are affected by periodontal disease and substantially interfere in the health-disease process, especially among the elderly, who are weaker from aging [17].

Given this prospect, national inquiries are important tools that aid in identifying the health conditions of the overall population [18]. In 2010, the National Oral Health Survey (SB Brasil 2010) was carried out in Brazil. It was considered the largest and most complex national survey on oral health [19] providing a sizable database representing the oral health conditions of the Brazilian population, including the elderly. Within the scope of primary healthcare and the Family Health Strategy (FHS) program, this survey has allowed decisions to be made on the basis of epidemiological data, thus strengthening specific actions aimed at meeting the needs inherent to this age group, with the support of health surveillance [20,21].

Aging impacts individual as well as contextual factors, and thus increases the potential for developing illnesses and the difficulty in gaining access to health services [22]. All of this is worsened by the lack of knowledge about the magnitude or the effect of inequality on one’s health, including the effects on oral health [23]. Therefore, the aim of the present study was to assess the potential influence of contextual as well as individual variables on the need for periodontal treatment of elderly Brazilian people.

## Material and Methods

The present study was conducted based on data from the National Oral Health Survey (SB Brasil 2010), as well as the contextual features of the municipalities assessed [19]. It was subject to analysis by the Universidade Federal de Mato Grosso do Sul (UFMS) Institutional Review Board, aiming at obtaining a favorable opinion regarding its development, and was approved under protocol #675.217.

SB Brasil 2010 was a nationwide epidemiological survey assessing different factors responsible for a scenario of worsening oral health. It was conducted using the probability cluster sampling technique, with a sample comprising 32 geographic domains: one for each capital city and one for the Federal District (27 domains), in addition to five for inland municipalities, one from each region of Brazil (North, Northeast, Central-West, Southeast and South) [19].

The databank from the 2010 National Oral Health Survey used in this study is available upon request—the data were obtained from The Brazilian Ministry of Health, a public database assessable upon request to the Oral Health Coordination of the Ministry of Health, at the following electronic address: http://dab.saude.gov.br/portaldab/ape_brasil_sorridente.php?conteudo=solicitacao_bd_sb2010. Parties interested in gaining access to the dataset must complete the following information: a) fill out the form containing the main data on the researcher, affiliated institution and research project; b) fill out and sign a commitment agreement containing the general regulations of the database. The filled out and signed form and commitment agreement must be sent by mail, fax or email (PDF format) to the following address: Coordenação Geral de Saúde Bucal–Ministério da Saúde/Departamento de Atenção Básica Adress: Edifício Premium Torre II–Sala 06 –Setor de administração Federal Sul–Quadra 2 –Lote 5/6 –CEP 70070–600 Email: cosab@saude.gov.br

This epidemiological survey assessed the periodontal conditions of the elderly, aged between 65 and 74 years (n = 7,619), according to the Community Periodontal Index (CPI) recommended by the World Health Organization (OMS). The need for clinical periodontal treatment was assessed based on data on dental calculus, shallow periodontal pockets (3–5 mm) and deep periodontal pockets (≥ 6 mm). The index was adapted to assess the individual prevalence of dental calculus, gingival bleeding and periodontal pockets in each dental arch sextant.

Index modification consisted of independently recording the presence or absence of the following four indicators in each sextant examined: gingival bleeding after probing, dental calculus, shallow periodontal pockets (3 mm to 5 mm) and deep periodontal pockets (6 mm or more). The elderly could be included in more than one category if they had at least one sextant with the presence of the indicator; for example, one sextant with both deep periodontal pockets and dental calculus. If index teeth were missing, all the remaining teeth of the sextant were examined, except the distal surface of the third molars [8]. The presence of two or more teeth and no indication of tooth extraction were considered a prerequisite for the inclusion of the sextant. Sextants with only one functioning tooth or without any teeth were excluded.

Although gingival bleeding does not require clinical treatment, its presence was a factor considered for analysis, because it is a condition causing alert and cautioning toward the promotion and raising of awareness on health matters. CPI adaptation is an important strategy, since conventional CPI assessment of the worst sextant score might fail to reveal the real prevalence of the conditions assessed. Data were collected by clinical examination under natural lighting, using an oral mirror and a ball-point probe, and conducted by teams consisting of a general dentist and a note taker [19].

The correlation between periodontal conditions and individual as well as contextual factors surveyed for the year of 2010 were assessed only in elderly people that were not edentulous (n = 3.926). The following contextual factors were analyzed: Municipal Human Development Index (MHDI), Gini Index and population coverage by the Family Health Strategy (FHS) program oral health teams. The MHDI is a geometrical measure assessing income, education level and longevity dimensions. It ranges from 0 to 1, where the higher the value, the better the social conditions [24].

The MHDI was grouped as follows: less than or equal to 0.7, and greater than 0.7, according to the UNDP atlas. The Gini Index is a statistical measure assessing income inequality; it ranges from 0 to 1, where 0 corresponds to absolute equality and 1 to absolute inequality [24]. The Gini Index was grouped according to the distribution of tertiles into: lower tertile, middle tertile and upper tertile.

The population coverage estimated by the Family Health Strategy (FHS) program oral health teams for the municipalities selected by the SB Brasil 2010 was used as an indicator of the offer and ease of access to primary dental care services [25]. Population coverage was grouped as follows: less than 25%, between 25% and 50% and greater than 50%.

The FHS contextual variable corresponds to the mean monthly number of primary oral health teams for every 1,000 families (about 3,000 individuals) in relation to the total population of the municipality in the year analyzed. The Brazilian Ministry of Health (BMH) establishes 50% of oral health coverage as the parameter, but we chose to stratify into less than 25%, from 25–50% and more than 50%, because 6,293 elderly people were under 50% coverage. The webpage of the performance index of the Brazilian Public Healthcare System was consulted to acquire data for 2010 for each municipality [25].

Data on individual factors were collected through the SB Brasil 2010, by individual interviews conducted at the respondents’ homes, with a structured questionnaire. The following information was gathered, composing the independent variables: sex, skin color, education level and family income. Education level was assessed by estimating the number of years of complete education (no failing grade), and was considered a continuous variable. Self-reported skin color was classified as white and nonwhite (black, mulatto and others), and monthly family income was reported and grouped as: up to R$ 1,500.00 and above R$ 1,500.00.

Correlations between periodontal conditions and individual as well as contextual factors were estimated by odds ratio (OR), with a significance level set at 5% and a 95% confidence interval (CI95%). Odds ratio values were obtained by multilevel logistic regression models with mixed effect models (random and fixed), random intercept and logit function (mixed effects), with Stata^TM^ software, version 13.0 (StataCorp., College Station, TX, USA).

## Results

The total sample of elderly people assessed by this study was 3,926 individuals; 58.1% were females, 61% reported an income no greater than R$ 1,500.00, and 48.3% self-reported as white (Table 1). The mean years of study was 6 (6 ± 4.6), and the mean number of teeth present was 14.3 (CI95% 14.04–14.58).

**Table 1:** Descriptive analysis of the contextual and individual variables and assessment of need for treatment (n = 3,926). Brasil, 2010.

Variable	n (%)
***Contextual***	
Gini Coefficient	
Lower tertile	1,163 (29.62)
Middle tertile	1,212 (30.87)
Upper tertile	1,551 (39.5)
MHDI	
≤ 0.7	351(9)
> 0.7	3,575 (91)
SB/FHS coverage	
< 25%	1,666 (42.5)
25%–50%	1,693(43.1)
>50%	567(14.4)
***Individual***	
Sex	
Male	1,645 (41.9)
Female	2,281 (58.1)
Self-reported skin color	
White	1,897 (48.3)
Nonwhite	2,029 (51.7)
Income	
< R$ 1,500.00	2,397 (61)
> R$ 1,500.00	1,529 (39)
***Need for treatment***	
Dental calculus	
Yes	2,590 (66)
No	1,336 (34)
Shallow periodontal pocket (3 mm to 5 mm)	
Yes	1,189 (30.3)
No	2,737 (69.7)
Deep periodontal pocket (6 mm or more)	
Yes	320 (8.2)
No	3,606 (91.8)

As regards the contextual determinants, 39.5% of the elderly lived in municipalities with a high Gini Index (third tertile), and 43.1% had coverage ranging from 25% and 50% by the Family Health Strategy (FHS) program oral health teams. Moreover, 93.5% of the elderly lived in municipalities with a high MHDI (> 0.7), as shown in Table 1.

Analysis of the contextual and individual variables, potentially associated with the need for periodontal treatment, revealed that individual factors were associated with all the outcomes assessed. The association between gingival bleeding and the female sex was reduced, thus making sex a protective factor and lowering the chances of presenting such an outcome in 13% of the population. Elderly people, self-reporting as nonwhite, presented a higher risk of gingival bleeding (OR = 1.32; CI95% 1.14–1.53), as shown in Table 2.

**Table 2:** Multilevel logistic regression of determinants in the presence of gingival bleeding. SB Brasil, 2010.

Variable	OR	CI95%	*p* value
*Contextual*			
Gini Index ^a^			
Middle tertile	0.74	0.54–1.00	0.05
Upper tertile	0.77	0.56–1.07	0.12
MHDI ^b^	0.94	0.65–1.34	0.73
SB/FHS coverage ^c^			
25%-50%	0.67	0.52–0.88	0.004
>50%	0.53	0.39–0.73	<0.001
*Individual*			
Sex ^d^	0.87	0.76–1.00	0.02
Self-reported skin color ^e^	1.32	1.14–1.53	<0.001
Income ^f^	0.83	0.72–0.97	0.40
Education	0.73	0.63–0.85	0.10

^a^ lower tertile = 1, middle tertile = 2 and upper tertile = 3.

^b^ ≤ 0.7 = 1; >0.7 = 2.

^c^ < 25% = 0; 25%-50% = 1; >50% = 2.

^d^ male = 0; female = 1.

^e^ white = 0; nonwhite = 1.

^f^ ≤ R$ 1,500 = 0; > R$ 1,500 = 1.

Missing data: income (159) and education (212).

Sextant excluded or not evaluated: sex 17 (2,514); sex 11 (2,297); sex 27 (2,559); sex 37 (2,211); sex 31 (632); sex 47 (2,201).

Of the individual factors analyzed, sex was also associated with a lower presence of dental calculus (OR = 0.86; CI95% 0.75–0.99) among women, thus reducing their chances of calculus. Skin color, self-reported as nonwhite (OR = 1.32; CI95%1.14–1.54), as well as level of education (OR = 0.77; CI95% 0.66–0.89), were risk factors for the presence of dental calculus. Women also presented a 31% smaller chance of presenting shallow periodontal pockets (OR = 0.69; CI95% 0.60–0.80). Self-reported skin color (OR = 1.27; CI95% 1.09–1.49) and level of education (OR = 0.86; CI95% 0.73–1.00) were individual factors also associated with shallow periodontal pockets, and acted as risk factors for nonwhite and undereducated elderly people. The individual variables associated with the presence of deep periodontal pockets were sex (OR = 0.58; CI95% 0.45–0.74), with women and education level showing smaller chances of needing treatment (OR = 0.58; CI95% 0.45–0.74, respectively), as shown in Table 3.

**Table 3:** Multilevel logistic regression of determinants in the presence of dental calculus, shallow periodontal pockets and deep periodontal pockets. SB Brasil, 2010.

Variable	Calculus			Shallow periodontal pocket (3 mm- 5 mm)			Deep periodontal pocket (≥ 6 mm)		
	OR	CI95%	*p* value	OR	CI95%	*p* value	OR	CI95%	*p* value
***Contextual***									
Gini Index ^a^									
Middle tertile	0.81	0.59–1.13	0.22	1.02	0.76–1.39	0.85	1.02	0.68–1.55	0.89
Upper tertile	0.93	0.66–1.33	0.71	0.74	0.53–1.03	0.07	0.64	0.41–1.00	0.06
MHDI ^b^	1.03	0.70–1.50	0.48	0.86	0.59–1.24	0.42	1.09	0.64–1.88	0.86
SB/FHS ^c^									
25%-50%	0.87	0.65–1.14	0.32	0.76	0.58–0.98	0.03	0.62	0.44–0.89	0.01
>50%	0.95	0.69–1.32	0.80	0.68	0.50–0.93	0.01	0.58	0.37–0.92	0.02
***Individual***									
Sex ^d^	0.86	0.75–0.99	<0.001	0.69	0.60–0.80	<0.001	0.58	0.45–0.74	<0.001
Skin color ^e^	1.32	1.14–1.54	<0.001	1.27	1.09–1.49	0.002	1.10	0.84–1.43	0.46
Family income ^f^	0.74	0.63–0.85	0.24	0.89	0.76–1.05	0.17	0.91	0.69–1.20	0.54
Education	0.77	0.66–0.89	<0.001	0.86	0.73–1.00	0.06	0.74	0.57–0.97	0.03

^a^ lower tertile = 1, middle tertile = 2 and upper tertile = 3.

^b^ ≤ 0.7 = 1; >0.7 = 2.

^c^ < 25% = 0; 25%-50% = 1; >50% = 2.

^d^ male = 0; female = 1.

^e^ white = 0; nonwhite = 1.

^f^ ≤ R$ 1,500 = 0; > R$ 1,500 = 1.

Missing data: income (159) and education (212).

Sextant excluded or not evaluated: sex 17 (2,514); sex 11 (2,297); sex 27 (2,559); sex 37 (2,211); sex 31 (632); sex 47 (2,201).

As for contextual determinants, a correlation was found between population coverage by the Family Health Strategy (FHS) program oral health teams and the presence of gingival bleeding (OR = 0.67; CI95% 0.52–0.88), shallow periodontal pockets (OR = 0.76; CI95% 0.58–0.98) and deep periodontal pockets (OR = 0.62; CI95% 0.44–0.89), thus making oral health team coverage a protective factor, and reducing the chance of an elderly person presenting with the need for treatment (Tables 2 and 3).

## Discussion

Periodontal condition assessment based on the SB Brasil 2010 group of 65- to 74-year-olds revealed that elderly people had few sextants presenting good conditions of examination, since sextants were excluded from 90.5% of the sample. There was a low prevalence of periodontal pockets among the elderly participating in the survey. Previous studies found similar results in different populations. In a nationwide study conducted by Morris et al. [26] in the United Kingdom, only 4% of elderly people presented with deep periodontal pockets. Borrell et al.[10] yielded similar results (6.3%) among North American elderly people.

The chances of needing periodontal treatment were higher among males, thus corroborating the results of previous studies, which found a correlation between sex and periodontal disease [27–30]. This difference between males and females could be a result of oral hygiene habits, the effort of seeking dental care services, and other factors, such as smoking [30]. Studies have found a higher prevalence of smoking habits among elderly males in Brazil [31,32] with smoking acting as a major risk factor for periodontal disease [7,30,33,34]. Additionally, 56.2% of women aged between 65 and 74 years, assessed by the SB Brasil 2010, were edentulous [15] in other words, a wide range of females were not subject to periodontal evaluation.

The need to improve oral health epidemiological indices in Brazil led to the inclusion of oral health teams in the Family Health Strategy (FHS) program, designed to improve access of the Brazilian population to dental care services [35] Analyses of the potential impact caused by the expansion of the Family Health Strategy (FHS) program oral health teams on increased access to oral health services and on the reduced need for treatment have made it possible to assess whether the insertion of oral health teams has provided effective changes to be made in the oral health of Brazilian elderly people, a group that has been given little priority by welfare programs throughout history.

Although gingival bleeding does not require clinical treatment, it is considered a condition causing alert and cautioning toward the promotion and raising of awareness on health matters [36]. Results of the present study revealed a correlation between the presence of gingival bleeding and coverage of the population by the Family Health Strategy (FHS) program oral health teams. Municipalities having this coverage for over 50% of the population had elderly people presenting with little prevalence of gingival bleeding. These outcomes could suggest a potential protective factor resulting from actions taken to promote and raise awareness on health matters, developed by the FHS program oral health teams [37] to restrain the development of disease, seeing that gingival bleeding may be considered one of the clinical signs of periodontal disease that could compromise dental supporting tissues, if not restrained.

The coverage estimated by the Family Health Strategy (FHS) oral health teams is viewed as a protective factor associated with two out of three clinical periodontal features used to determine the need for periodontal treatment (shallow and deep periodontal pockets). These findings suggest a potential positive effect on the periodontal health of elderly people, resulting from greater population coverage by the Family Health Strategy (FHS) program oral health teams, and, consequently, from greater supply and access to primary dental care services. These conclusions reinforce the importance of increasing FHS coverage, so as to reduce inequality in oral health.

Self-reported nonwhite skin color was associated with the need for periodontal treatment. This was especially true of elderly people self-reporting as nonwhite, who presented higher chances of needing treatment, as previously demonstrated [10,27,28,29,38,39]. Other studies also revealed inequality among ethnic groups in Brazil [28,40] and difficulty faced by nonwhite elderly people in having access to dental care services [40]. Other hypotheses to explain this correlation could be associated with the influence of the most frequent findings reported for nonwhites, namely: poor education, poor job conditions, physical and psychological stress due to racial prejudice and consequent excessive consumption of alcohol and cigarettes [38].

Education level was associated with the presence of dental calculus and deep periodontal pockets, thus acting as a risk factor for undereducated elderly people, which could be associated with the tendency of undereducated people to have less healthy habits and rarely seek health care services [39,41,42], thus implying greater treatment needs. Previous studies [29,39,43] have found a correlation between income and periodontal disease; however, this does not corroborate the results of the present study. After adjusting for contextual and other individual features, no association was found between the need for periodontal treatment and elderly people’s income, thereby suggesting, in this case, a more significant influence of all the other individual and contextual factors, or else a consequence of the choice of variables assessed. This is because the influence of contextual and individual determinants could vary according to the outcomes used to analyze a person’s oral health conditions [39,44–46].

The cross-sectional nature of the present study does not allow a causal relationship to be established between the correlations found herein. This restricts the interpretation of results and reinforces the need for further studies with a specific design to provide in-depth analysis of the impact of contextual and individual determinants on the need for periodontal treatment among elderly people. Nevertheless, the information provided by the present study can serve to guide oral health action planning, which is key and critical in the context of primary healthcare (represented herein by the Family Health Strategy [FHS] program), given the characteristics inherent to the process of revamping the health care model currently effective in Brazil.

## Conclusions

This study showed evidence of the sociocontextual as well as individual sociodemographic characteristics influencing periodontal treatment needed by elderly Brazilians, based on the clinical features of periodontal disease. The results suggest the existence of inequality related to periodontal treatment needs among Brazilian elderly people, especially in regard to sex and ethnicity, in addition to a potential positive impact from the expansion of the Family Health Strategy (FHS) program oral health teams on reducing periodontal treatment needs among the elderly population.

## Supporting Information

S1 File SB Brazil Codebook. The codebook of the databank from the 2010 National Oral Health Survey.
(XLS)

## References

[1] Carvalho JAM, Garcia RA. The aging process in the Brazilian population: a demographic approach. Cad. Saude Publica 2003;19(3): 725–733. 12806475 10.1590/s0102-311x2003000300005

[2] Carvalho JAM, Rodríguez-Wong LL. The changing age distribution of the Brazilian population in the first half of the 21^st^ century. Cad. Saude Publica, 2008; 24(3): 597–605. 18327447 10.1590/s0102-311x2008000300013

[3] Vasconcelos AMN, Gomes MMF. Demographic transition: the Brazilian experience. Epidemiol. Serv. Saúde. 2012; 21(4)539–548.

[4] Harford J. Population ageing and dental care. Community Dent Oral Epidemiol. 2009; 37:97–103. 10.1111/j.1600-0528.2008.00441.x 18782331

[5] Petersen PE, Bourgeois D, Ogawa H, Estupinan-Day S, Ndiaye C. The global burden of oral disease and risks to oral health. Bulletin of the World Health Organization. 2005; 3:661–669. PMC2626328 16211157

[6] Watt RG, Petersen PE. Periodontal health through public health–the case for oral health promotion. Periodontol 2000. 2012; 60(1):147–155. 10.1111/j.1600-0757.2011.00426.x 22909112

[7] Petersen PE, Baehni PC. Periodontal health and global public health. Periodontol 2000. 2012; 60(1): 7–14. 10.1111/j.1600-0757.2012.00452.x 22909103

[8] Sheiham A, Netuveli GS. Periodontal diseases in Europe. Periodontol 2000 2002; 29:104–121. 12102705 10.1034/j.1600-0757.2002.290106.x

[9] Corbet EF, Zee KY, Lo E. Periodontal diseases in Asia and Oceania. Periodontol 2000 2002; 29(1): 122–152. 12102706 10.1034/j.1600-0757.2002.290107.x

[10] Borrell LN, Burt BA, Neighbors HW, Taylor GW. Social factors and periodontitis in an older population. Am J Public Health 2004;94:748–754. 15117695 10.2105/ajph.94.5.748 PMC1448332

[11] Aida J, Ando Y, Akhter R, Aoyama H, Masui M, Morita M. Reasons for permanent tooth extractions in Japan. J Epidemiol. 2006; 16(5):214–219. 16951541 10.2188/jea.16.214 PMC7683702

[12] Hiramatsu DA, Tomita NE, Franco LJ. Tooth loss and the image of the dentist in a group of senior citizens.Cien. Saude Colet. 2007;12(4):1051–1056. 17680163 10.1590/s1413-81232007000400026

[13] Jovino-Silveira RC, Caldas AF JR, Souza EH, Gusmão ES. Primary reason for tooth extraction in a Brazilian adult population. Oral Health Prev Dent. 2005;3:151–7. 16355648

[14] Peres MA, Barbato PR, Reis SCGB, De Morais Freitas CHS, Antunes JLF. Tooth loss in Brazil: analysis of the 2010 Brazilian Oral Health Survey. Rev. Saúde Publ, 2013; 47(suppl. 3):78–89. 10.1590/s0034-8910.2013047004226 24626584

[15] Antunes JLF, Pegoretti T, Andrade FP, Junqueira SR, Frazão P, Narvai PC. Ethnic disparities in the prevalence of dental caries and restorative dental treatment in Brazilian children. Int Dent J. 2003;53(1):7–12. 12653333 10.1111/j.1875-595x.2003.tb00649.x

[16] Li X, Kolltveit KM, Tronstad L, Olsen I. Systemic diseases caused by oral infection. Clin Microbiol Rev. 2000;13(4):547–558. 11023956 10.1128/cmr.13.4.547-558.2000 PMC88948

[17] Veras R. Population aging today: demands, challenges and innovations. Rev. Saúde Publi. 2009; 43(3):548–554. 10.1590/s0034-89102009000300020 19377752

[18] Martins AMEBL, Melo FS, Fernandes FM, Boa Sorte JA, Coimbra LGA, Batista RC. National epidemiological surveys on conditions of oral health, Brazil. Unimontes Cient. 2005;7(1)55–66.

[19] Brazil Ministry of Health. Department of Health Care. Department of Primary Care. National Coordination of Oral Health. SB Brazil 2010: National Oral Health Survey: Main results, 2011.

[20] Teixeira CFS. Epidemiology and health planning. Cien. Saude Colet. 1999;4(2): 287–303.

[21] Barbato PR, Nagano HCM, Zanchet FN, Boing AF, Peres MA. Tooth loss and associated socioeconomic, demographic, and dental-care factors in Brazilian adults: an analysis of the Brazilian Oral Health Survey, 2002–2003. Cad. Saude Publica 2007;23(8):1803–1814. 17653398 10.1590/s0102-311x2007000800007

[22] Rodrigues NO, Neri AL. Social, individual and programmatic vulnerability among the elderly in the community: data from the FIBRA Study conducted in Campinas, São Paulo, Brazil. Cien. Saude Colet. 2012; 17(8):2129–2139. 22899153 10.1590/s1413-81232012000800023

[23] Shi L, Tsai J, Kao S. Public health, social determinants of health and public policy. J Med. Sci. 2009;29(2):43–59.

[24] United Nations Development Programme. Atlas of Human Development in Brazil: 1991–2010. 2013.

[25] Brazil. Ministry of Health. DATASUS. Health information. Coverage of oral health teams, 2010.

[26] Morris AJ, Steele J, White DA. The oral cleanliness and periodontal health of UK adults in 1998. Br Dent J 2001: 191: 186–192. 11551090 10.1038/sj.bdj.4801135

[27] Albandar JM. Global risk factors and risk indicators for periodontal diseases. Periodontol 2000 2002;29(1):177–206. 12102708 10.1034/j.1600-0757.2002.290109.x

[28] Haas AN, Gaio EJ, Oppermann RV, Rösing CK, Albandar JM, Susin C. Pattern and rate of progression of periodontal attachment loss in an urban population of South Brazil: a 5‐years population‐based prospective study. J Clin. Periodontol. 2012;39(1):1–9. 10.1111/j.1600-051X.2011.01818.x 22093104

[29] Vettore MV, Marques RAA, Peres MA. Social inequalities and periodontal disease: multilevel approach in SBBrasil 2010 survey. Rev Saúde Publi. 2013;47(suppl.3): 29–39. 10.1590/s0034-8910.2013047004422 24626579

[30] Genco RJ, Borgnakke WS. Risk factors for periodontal disease. Periodontol 2000 2013; 62(1):59–94. 10.1111/j.1600-0757.2012.00457.x 23574464

[31] Peixoto SV, Firmo JOA, Lima-Costa MF. Health conditions and smoking among older adults in two communities in Brazil (The Bambuí and Belo Horizonte Health Surveys). Cad. Saúde Pública 2006;22(9)1925–1934. 16917590 10.1590/s0102-311x2006000900024

[32] Zaitune MPA, Barros MBA, Lima MG, César CLG, Carandina L, Goldbaum M, et al Factors associated with smoking in the elderly: a health survey in São Paulo (ISA-SP). Cad. Saúde Pública 2012;28(3):583–595. 22415190 10.1590/s0102-311x2012000300018

[33] Klinge B, Norlund A. A socio‐economic perspective on periodontal diseases: a systematic review. J Clin. Periodontol. 2005;32,(s6):314–325. 16128846 10.1111/j.1600-051X.2005.00801.x

[34] Gomes R, Nascimento EF, Araujo FC. Why do men use health services less than women? Explanations by men with low versus higher education. Cad Saude Publica 2007; 23(3):565–574. 17334571 10.1590/s0102-311x2007000300015

[35] Baldani MH, Narvai PC, Antunes JLF. Dental caries and socioeconomic conditions in the State of Paraná, Brazil,1996. Cad Saude Publica 2002; 18(3): 755–763. 12048601 10.1590/s0102-311x2002000300024

[36] Antunes JLA, Peres MA, Frias AC, Crosato EM, Biazevic MGH. Gingival health of adolescents and the utilization of dental services, state of São Paulo, Brazil. Rev. Saúde Pública 2008;42(2):191–9. 18372971 10.1590/s0034-89102008000200002

[37] Tesser CD, Garcia AV, Argenta CE, Vendruscolo C. Conceptions of Health Promotion What permeate the ideology teams of the Family Health Strategy in Florianópolis. R. Saúde públ. Santa Cat. 2010; 3(1).

[38] Peres MA, Antunes JLF, Boing AF, Peres KG, Bastos JLD. Skin colour is associated with periodontal disease in Brazilian adults: a population-based oral health survey. J Clin. Periodontol. 2005;34:196–201. 10.1111/j.1600-051X.2006.01043.x 17257159

[39] Bastos JL, Boing AF, Peres KG, Antunes JL, Peres MA. Periodontal outcomes and social, racial and gender inequalities in Brazil: a systematic review of the literature between 1999 and 2008. Cad Saude Publica 2011; 27(Suppl 2):S141–53. 21789408 10.1590/s0102-311x2011001400003

[40] Souza EHA, Oliveira PAP, Paegle AC, Goes PSA. Race and the use of dental health services. by the elderly. Cien Saude Colet 2012; 17(8):2063–2070. 22899147 10.1590/s1413-81232012000800017

[41] Moreira RS, Nico LS, Tomita NE, Ruiz T. Oral health of Brazilian elderly: a systematic review of epidemiologic status and dental care access. Cad Saude Publica 2005; 21(6)1665–1675. 16410850 10.1590/s0102-311x2005000600013

[42] Ferreira CO, Antunes JLF, Andrade FB. Factors associated with the use of dental services by elderly Brazilians. Rev. Saúde Publi. 2013; 47(3):90–97. 10.1590/s0034-8910.2013047004721 24626585

[43] Sabbah W, Tsakos G, Chandola T, Sheiham A, Watt RG. Social Gradients in Oral and General Health. J Dent. Res. 2007; 86(10): 992–996. 17890677 10.1177/154405910708601014

[44] Steele J, Shen J, Tsakos G, Fuller E, Morris S, Watt R, et al The Interplay between Socioeconomic Inequalities and Clinical Oral Health. J Dent. Res. 2015; 94(1):19–26. 10.1177/0022034514553978 25344336

[45] Sabbah W, Sheiham A, Bernabé E. Income inequality and periodontal diseases in rich countries: an ecological cross-sectional study. Int. Dent. J 2010;60(5):370–4. 21141210

[46] Celeste RK, Fritzell J, Nadanovsky P. The relationship between levels of income inequality and dental caries and periodontal diseases. Cadernos de Saúde Pública 2011; 27 (6): 1111–1120. 21710008 10.1590/s0102-311x2011000600008

